# Probing the Increased Virulence of Severe Acute Respiratory Syndrome Coronavirus 2 B.1.617 (Indian Variant) From Predicted Spike Protein Structure

**DOI:** 10.7759/cureus.16905

**Published:** 2021-08-05

**Authors:** Houssein Hajj-Hassan, Kassem Hamze, Fadi Abdel Sater, Nadeem Kizilbash, Hassan M Khachfe

**Affiliations:** 1 Department of Biological and Chemical Sciences, International University of Beirut, Beirut, LBN; 2 Laboratory of Molecular Biology and Cancer Immunology (Covid-19 Unit), Lebanese University, Beirut, LBN; 3 Department of Medical Laboratory Technology, Northern Border University, Arar, SAU; 4 Lebanese Institute for Biomedical Research and Applications (LIBRA), Lebanese International University, Beirut, LBN

**Keywords:** covid-19, homology modeling, mutations, protein structure prediction, energy minimization, pandemic, variant

## Abstract

Coronavirus disease 2019 (COVID-19), caused by severe acute respiratory syndrome coronavirus 2 (SARS-CoV-2), has led to an outbreak of a pandemic worldwide. The spike (S) protein of SARS-CoV-2, which plays a key role in the receptor recognition and cell membrane fusion process, is composed of two subunits, S1 and S2. The S1 subunit contains a receptor-binding domain that recognizes and binds to the host receptor angiotensin-converting enzyme 2 (ACE2), while the S2 subunit mediates viral cell membrane fusion with the cell membrane and subsequent entry into cells. Mutations in the spike protein (S) are of particular interest due to their potential for reduced susceptibility to neutralizing antibodies or increasing the viral transmissibility and infectivity. Recently, many mutations in the spike protein released new variants, including the Delta and Kappa ones (known as the Indian variants). The variants Delta and Kappa are now of most recent concern because of their well-increased infectivity, both a spin-off of the B.1.617 lineage, which was first identified in India in October 2020. This study employed homology modeling to probe the potential structural effects of the mutations. It was found that the mutations, Leu452Arg, Thr478Lys, and Glu484Gln in the spike protein increase the affinity for the hACE2 receptor, which explains the greater infectivity of the SARS-Cov-2 B.1.617 (Indian Variant).

## Introduction

The severe acute respiratory syndrome coronavirus 2 (SARS-CoV-2) causes the COVID-19 pandemic, which, for more than one year, has been associated with a record number of cases and deaths [1]. Numerous strategies to fight this pandemic by vaccines or non-pharmaceutical interventions (e.g., forced lock-downs, masking up, hand washing, social distancing, etc.) have been lagged by the emergence of SARS-CoV-2 variants of concern (VOC). These variants harbor mutations that confer increased transmissibility (and death cases as a consequence) or immune evasion [2]. Many variants of concern and variant of interest have appeared in different countries, with combinations of mutations and deletions in the receptor-binding domain (RBD) and N-terminal domain of S protein. The variant Alpha “B.1.1.7” emerged in the United Kingdom, the variant Beta “B.1.351” in South Africa, the variants Gamma “P.1” and Zeta “P.2” in Brazil, the variant Epsilon “B.1.427/B.1.429” in California (USA), the variant Lambda "C.37" in Peru, and recently the variants Delta “B.1.617.2” and Kappa “B.1.617.1” in India [3-7].

Mutations in the spike protein (S) are of particular interest due to their potential for reduced susceptibility to neutralizing antibodies provoked by vaccination or prior infection. The spike protein is 1,273 amino acids long with two important functional regions: the N-terminal region (S1) (amino acids (aa) 14-682) responsible for viral attachment to target cells via the angiotensin-converting enzyme 2 (ACE2) receptor, and the C-terminal region (S2) (aa 686-1273) responsible for membrane fusion and cell entry [8]. Before fusion, S1 is cleaved from S2 in the cleavage region (aa 682- 685).

Antibodies against the ACE2 binding domain of S1 (RBD, aa 319-541) are considered critical in neutralizing novel coronavirus (nCoV) [9,10]. Because of the important functional and antigenic properties of RBD, structural changes in this domain deserve special attention. The sensitivity to antibody neutralization varies with the viral variant. The variant Alpha seems to be more sensitive to neutralization than Beta. The RBD mutation N501Y, which increases affinity to ACE2 and is present in the Alpha, Beta, and Gamma variants [11], does not have any effect on post-vaccine serum neutralization. It has been suggested that the other mutations in Alpha do not result in immune evasion of linear epitopes [12]. Mutations in the Beta and Gamma variants, including E484K and K417N/T, are of high concern since they partly compromise neutralization generated by previous infection or vaccination or affect viral stability [4, 13-14].

The L425R mutation, present in the variants Epsilon, Delta, and Kappa, reduces the spike protein reactivity with the virus-neutralizing antibodies and sera from convalescent patients [4, 15-16]. Moreover, it was found that the L452R mutation strengthens virus-cell attachment and, thus, increases infectiousness [17]. The variants Delta and Kappa have recently spread to several countries from India. They share an L452R mutation on the spike protein, which may be associated with increased transmissibility of the virus.

The variant kappa contains two mutations in the RBD, L452R, and E484Q, which had never been observed together before the emergence of this variant. The E484Q mutation is known to participate in the partial immune escape post-infection and post-vaccination and is responsible for resistance to certain monoclonal antibodies. Mutations at this same position (E484K) have been observed in the variants Gamma and Zeta, as well as in the variant Beta [18, 19]. 

In the Delta variant, the mutations found in its RBD are L452R and T478K. However, it does not have the E484Q mutation. This variant seems to be around 60% more transmissible than the already highly infectious Alpha variant; it is expected to rapidly outcompete other variants and become the dominant circulating variant over the coming months [20]. T478K mutation has appeared and risen in frequency since January 2021, predominantly in Mexico and the USA in variant B.1.1.519. An *in-silico* molecular dynamics study on the protein structure of spike has predicted that the T478K mutation may significantly alter the electrostatic surface of the protein, and therefore, the interaction with ACE2, drugs, or antibodies, and that the effect can be increased if combined by other co-occurring spike mutations [21].

In the present study, we have focused on the Delta and Kappa variants and utilized homology modeling (HM) simulations of S protein trimer to assess its dynamic behavior in terms of conformational stability as well as the interaction of isolated viral RBD in complex with human ACE2 to probe the specific interactions emanating from the double mutations (L452R/E484Q in Kappa and L452R/T478K in Delta) in the RBD-ACE2 complexes.

## Materials and methods

The template structure of SARS-Cov-2 S protein (S) in complex with angiotensin-converting enzyme 2 was downloaded from the Protein Data Bank (ID# 7DF4) [22]. The variant mutations were identified from the GISAID (GISAID Initiative, Munich, Germany) database [23]. Mutations, simulations, and molecular dynamics (energy minimization using the program included forcefields) were performed using the SwissPdb Viewer (Swiss Institute of Bioinformatics, Lausanne, Switzerland) [24] and the YASARA servers [25] using the following sequence: 1) The native structure is loaded to the application (SwissPdp Viewer (SPDBV) or YASARA); 2) one of the amino acids is mutated (or “swapped”) using the “Mutate” (in SPDBV) or “Swap” (in YASARA) functions; 3) like in YASARA, SPDBV initially gives the rotamer confirmation (set of side-chain allowed angles) based on the wild type side-chain orientation, with some corrections in SPDBV relative to the allowed space. However, since the mutant amino acid may end up in another side-chain conformation, SPDBV - unlike YASARA - allows to check other side-chain rotamers so that conflicting ones are disregarded; 4) the allowed structures are sequentially energy minimized, and the best structure (lowest “possible” energy) is selected; 5) the side chain is rechecked for any conflicts; 6) steps 1-5 are repeated for the subsequent amino acid. All pictures were produced using the YASARA application.

## Results

**Figure 1:**
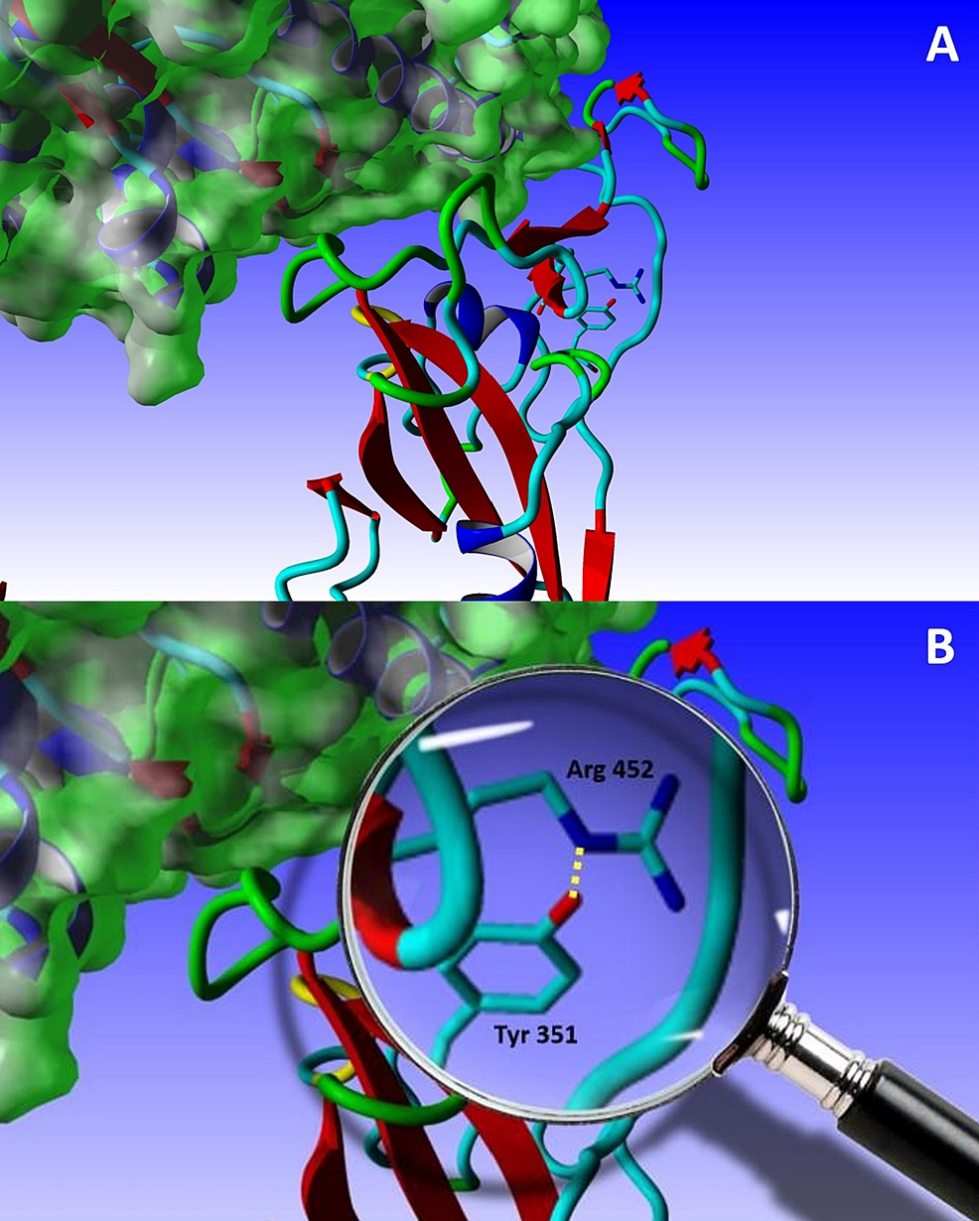
The L452R mutation (A) One of the energetically allowed rotamers of the L452R mutations. (B) A close-up showing the potentially-formed H-bond between Nε of Arg 452 and the hydroxyl hydrogen of Tyr 351. S is shown in tubes, whereas angiotensin-converting enzyme 2 (ACE2) is shown with the Van der Waals (VdW) surface in green.

Figure 1 shows the leucine-to-arginine mutation at position 452. Several rotamers were examined, and only the sterically allowed ones were further explored via energy minimization.

**Figure 2:**
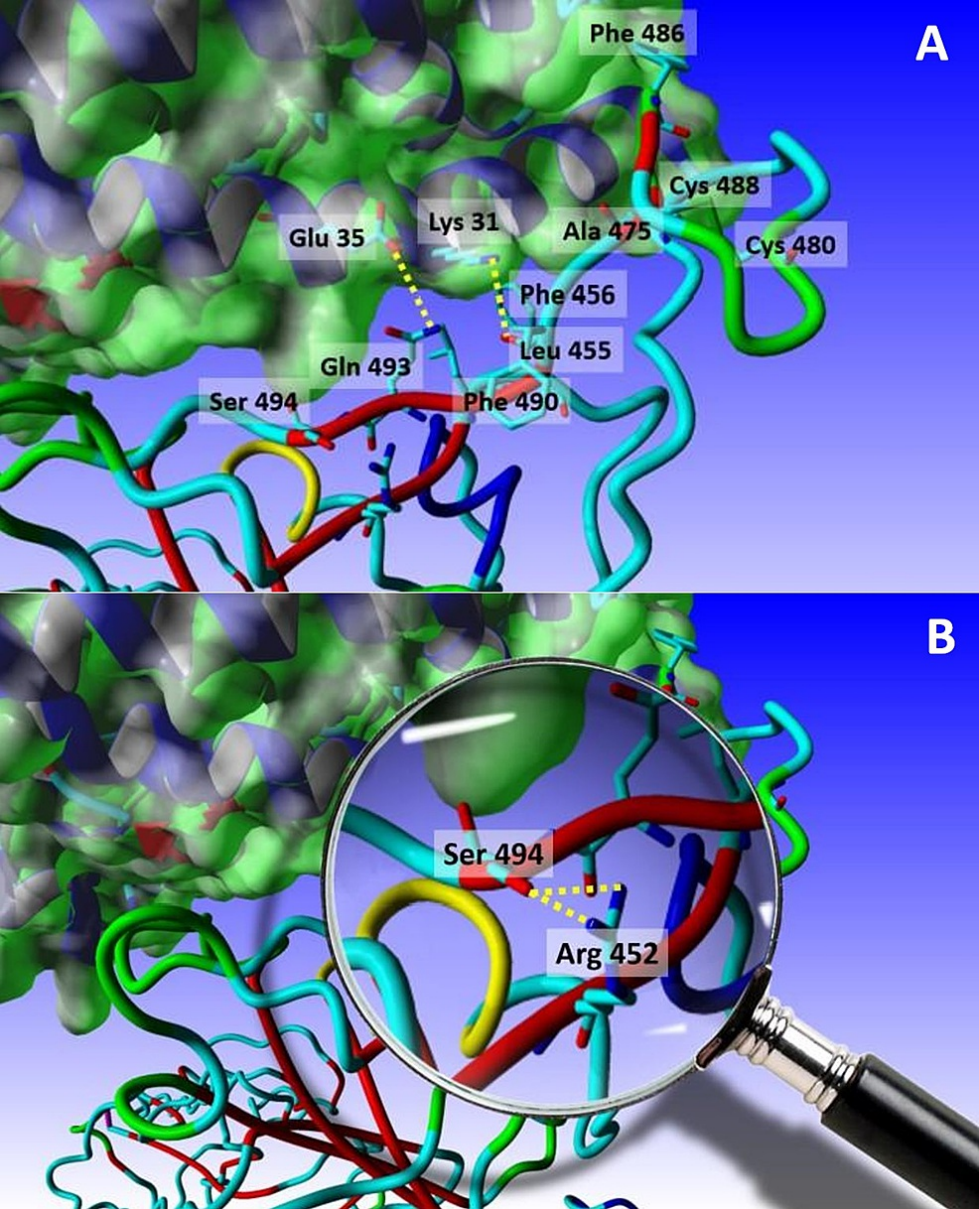
The interactions of Arg 452 (A) The receptor-binding motif (RBM), with the critical residues labeled. (B) A close-up showing the potentially-formed H-bond between NH1 and/or NH2 of Arg 452 and the hydroxyl hydrogen of Ser 494. S is shown in tubes, whereas angiotensin-converting enzyme 2 (ACE2) is shown with the Van der Waals (VdW) surface in green.

Figure 2 displays the potential interactions Arg 452 makes in the critical area of the receptor-binding motif.

**Figure 3:**
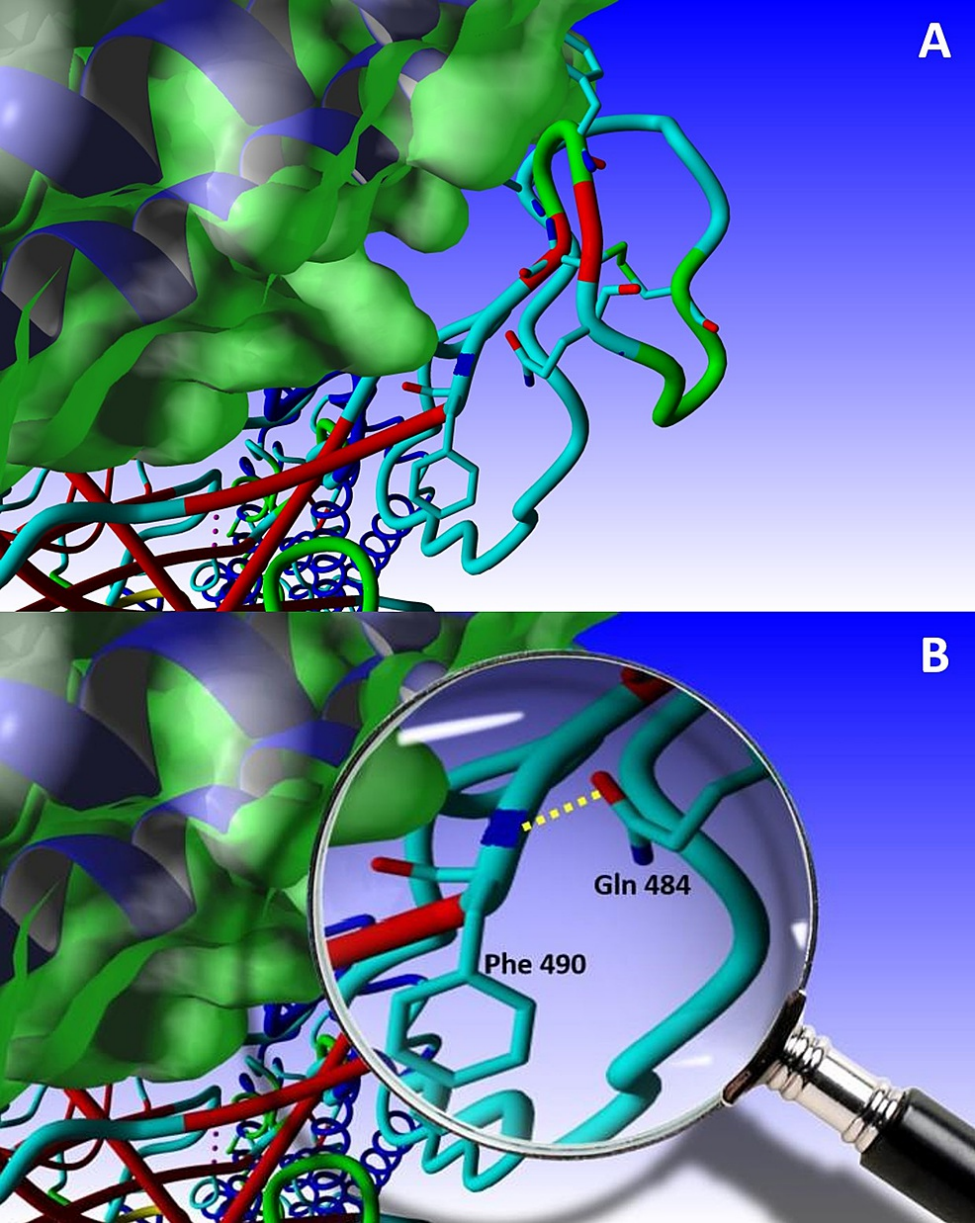
The E484Q mutation (A) The mutation of Glu 484 by Gln 484, and its position in the receptor-binding motif (RBM). (B) A close-up showing the potentially-formed H-bond between the side chain carbonyl of Gln 484 and the backbone amide hydrogen of Phe 490. S is shown in tubes, whereas angiotensin-converting enzyme 2 (ACE2) is shown with the Van der Waals (VdW) surface in green.

Figure 3 presents the glutamate-to-glutamine mutation at position 484. The newly mutated residue potentially H-bonds with the backbone nitrogen of Phe 490.

**Figure 4:**
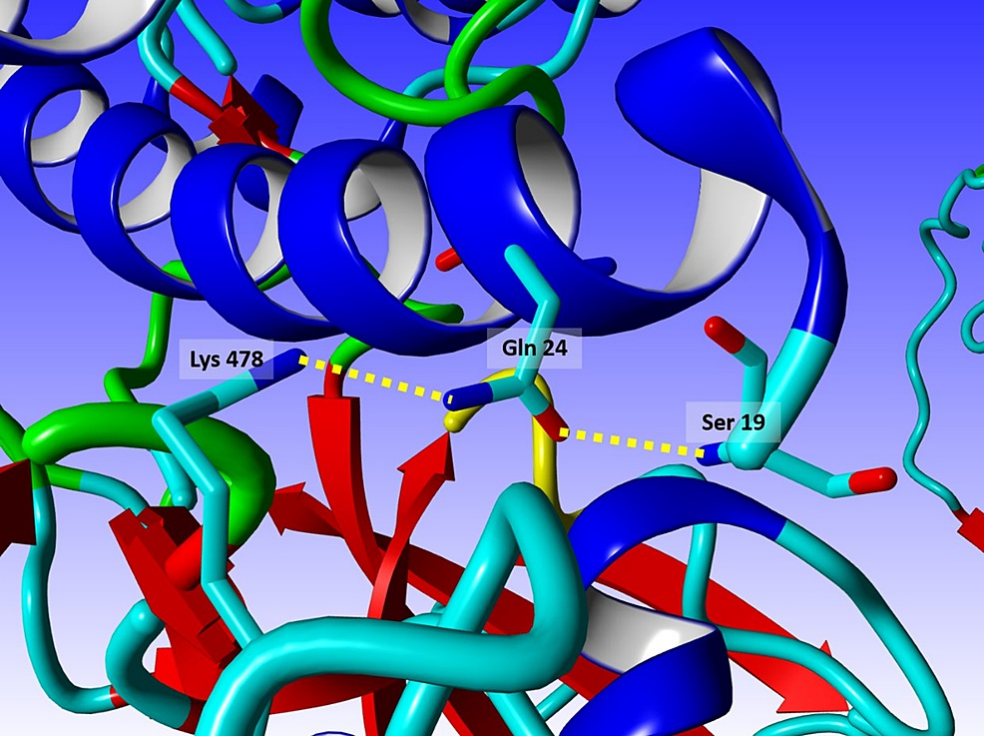
The T478K mutation The mutant T478K seems to stabilize the receptor binding by forming a strong H-bond with Gln 24 of angiotensin-converting enzyme 2 (ACE2). The native Thr 478 has no interaction, neither intra- nor inter-molecular, with its surrounding. The current mutation brings about more interaction between the receptor-binding motif (RBM) and ACE2.

Figure 4 exhibits the threonine-to-lysine mutation at position 478. Several of its rotamers were examined, along with those of Gln 24 of ACE2, and only the sterically allowed ones were further explored via energy minimization.

## Discussion

L452R

Mutating the leucine residue at position 452 to an arginine seems at first to have no structural/functional effect, given that it occurs in an otherwise solvent-exposed region in the RBD and on the surface of an amphipathic β-sheet. Moreover, most of the rotamers (28 in total) of the newly-mutated amino acid residue expose the solvent to the surface of the protein or hydrogen-bond to neighboring residues. Out of many, two such interactions are worth discussing.

For rotamers 4, 5, and 22, the Nε H-binds to the hydroxyl H of tyrosine 351 in an opposing loop in the core of the RBD. This interaction stabilizes the somewhat unstable loop - unlike the other one that is hinged by a disulfide bridge between cysteine 480 and cysteine 488 - which at the same time keeps the charged end towards the outside, thus contributing to the hydrophilic surface of the protein (Figure 1). On the other hand, for rotamers 1 and 9, the hydrogen atoms of NH1 and NH2 respectively bind to the carbonyl oxygen of serine 494. Due to its position in the β-strand of the receptor-binding motif (RBM) where it is directly in contact with ACE2, Ser494 is present close to Gln493 (which H-bond with Glu35 of ACE2) and Phe490, which is in the vicinity of Leu455 (which H-bond with Lys31 of ACE2), Phe456, Ala475, and Phe486 (Figure 2). All of these have been reported to be absolutely critical in the RBM and the interaction between the spike proteins and ACE2 [26]. This interaction between Arg452 and Ser495 not only protects the amphipathicity of the two-strand sheet in the RBM, but also helps in stabilizing the binding motif responsible for both recognition and binding, thus possibly explaining the increased affinity of the spike proteins - and therefore the virus - to its receptor, which explains the faster and increased pathogenicity observed due to this mutation.

E484Q

The mutation of the glutamate residue at position 484 with glutamine is particularly interesting. All but one of the 15 rotamers of the newly swapped residue have no structural problems and are therefore quite stable. Yet, only two of them form hydrogen bonds with neighboring residues, with one of these interactions occurring with the amide of the previously mentioned critical amino acid, Phe490 (Figure 3). This is particularly of interest since it adds to the stability of the loop already stabilized by the disulfide bond, and hence, immobilizes the rest of the critical amino acids found in the RBM, thus potentially increasing the affinity of the spike protein to its ACE2 receptor.

T478K

The 478 doesn’t seem to have any interaction, neither intra- nor inter-molecular, with the surrounding amino acids. Being on the surface of the RBM, it would definitely contribute to the solubility and surface polarity of the spike protein, but with no specific bonding effects. The mutant T478K appears to stabilize the receptor binding by forming a strong H-bond with Gln 24 of ACE2. Being on the surface of the protein, many of the different rotamers of the mutated Lysine allow for the polarity of that local to be preserved, yet one of them makes a specific interaction with Gln 24, which in turn, and upon a nice rotamer auto-rearrangement, creates another stabilizing H-bond with Ser 19 of ACE2. The current mutation brings about more interaction between the RBM and ACE2, all while maintaining the polarity of the surface.

Variants bearing two of the above-mentioned mutations (L452R/E484Q in Kappa variant or L452R/T478K in Delta variant) do not seem to have structural changes compared to the wild-type spike protein, yet, their effect on the receptor-binding function seems to be enhanced. Therefore, it’s more favorable to stabilize the spike structure and/or its interaction with the ACE2 receptor. This is reflected in the increased virulence and pathogenicity of the variant Delta. A large body of evidence indicates that the Delta variant (bearing the L452R/T478K double mutation) is the most and fastest-spreading variant in Europe and many countries and it will be the dominant one in the coming weeks. However, the Kappa variant (bearing the L452R/E484Q double mutation) or known as B.1.167.1, has raised red flags and led to widespread gene surveillance to look for its prevalence and spread. This may explain, somehow, the increased infectivity and virulence of the variant Delta in young persons, who express lower numbers of ACE2 receptors [27]. It has also been suggested that children have ACE2 receptors with a lower affinity for SARS-CoV-2 and a different distribution across body sites, making the entry of SARS-CoV-2 into cells more difficult [28].

Limitations

It should be noted that, while the above approach took into consideration all the “physically” necessary constraints (bond angles, bond lengths, steric hindrances, chemical and/or physical complementarities), it remains an *in-silico* methodology that lacks biophysical structural confirmation. Also, the absence or scarcity of point-mutation studies - and its comparison thereof with the wildtype - limits the biochemical understanding of the structure-function relationship in the corresponding variants.

## Conclusions

It is clear that the above-mentioned point mutations not only contribute greatly to the virulence of the SARS-Cov-2 variant Delta but also increase its pathogenicity manyfold. For example, the mutations Leu452Arg and Thr478Lys have an effect on the increased affinity of the spike proteins for the ACE2 receptor, which explains the greater pathogenicity of the variant. Also, this may explain, somehow, the increased infectivity and virulence of the variant Delta in young persons who express lower numbers of ACE2 receptors. It has also been suggested that children have ACE2 receptors with a lower affinity for SARS-CoV-2 and a different distribution across body sites, making the entry of SARS-CoV-2 into cells more difficult. In the absence of any experimentally determined structures of any of the variants, the *in-silico* approach provides one way to understand the structure-function relationship of the disease.

## References

[1] (2021). World Health Organization (WHO) Coronavirus Dashboard. https://www.who.int/emergencies/diseases/novel-coronavirus-2019?adgroupsurvey={adgroupsurvey}&gclid=CjwKCAjw87SHBhBiEiwAukSeUaXwwPF41319QhxMOemJ5-p8wCIar_VT6s-Bvg0bo9rcdIdODwC8xxoCkngQAvD_BwE.

[2] Plante JA, Mitchell BM, Plante KS, Debbink K, Weaver SC, Menachery VD (2021). The variant gambit: COVID-19's next move. Cell Host Microbe.

[3] Walls AC, Park YJ, Tortorici MA, Wall A, McGuire AT, Veesler D (2020). Structure, function, and antigenicity of the SARS-CoV-2 spike glycoprotein. Cell.

[4] Greaney AJ, Loes AN, Crawford KH, Starr TN, Malone KD, Chu HY, Bloom JD (2021). Comprehensive mapping of mutations in the SARS-CoV-2 receptor-binding domain that affect recognition by polyclonal human plasma antibodies. Cell Host Microbe.

[5] Ku Z, Xie X, Davidson E (2021). Molecular determinants and mechanism for antibody cocktail preventing SARS-CoV-2 escape. Nat Commun.

[6] Tegally H, Wilkinson E, Giovanetti M (2021). Detection of a SARS-CoV-2 variant of concern in South Africa. Nature.

[7] Frampton D, Rampling T, Cross A (2021). Genomic characteristics and clinical effect of the emergent SARS-CoV-2 B.1.1.7 lineage in London, UK: a whole-genome sequencing and hospital-based cohort study. Lancet Infect Dis.

[8] Buss LF, Prete CA Jr, Abrahim CM (2021). Three-quarters attack rate of SARS-CoV-2 in the Brazilian Amazon during a largely unmitigated epidemic. Science.

[9] Nascimento VA, Corado AL, Nascimento FO (2021). Genomic and phylogenetic characterisation of an imported case of SARS-CoV-2 in Amazonas State, Brazil. Mem Inst Oswaldo Cruz.

[10] Sabino EC, Buss LF, Carvalho MP (2021). Resurgence of COVID-19 in Manaus, Brazil, despite high seroprevalence. Lancet.

[11] Xie X, Liu Y, Liu J (2021). Neutralization of SARS-CoV-2 spike 69/70 deletion, E484K and N501Y variants by BNT162b2 vaccine-elicited sera. Nat Med.

[12] Shen X, Tang H, McDanal C (2021). SARS-CoV-2 variant B.1.1.7 is susceptible to neutralizing antibodies elicited by ancestral spike vaccines. Cell Host Microbe.

[13] Wibmer CK, Ayres F, Hermanus T (2021). SARS-CoV-2 501Y.V2 escapes neutralization by South African COVID-19 donor plasma. Nat Med.

[14] Thomson EC, Rosen LE, Shepherd JG (2021). Circulating SARS-CoV-2 spike N439K variants maintain fitness while evading antibody-mediated immunity. Cell.

[15] Li Q, Wu J, Nie J (2020). The Impact of Mutations in SARS-CoV-2 Spike on Viral Infectivity and Antigenicity. Cell.

[16] Starr TN, Greaney AJ, Dingens AS, Bloom JD (2021). Complete map of SARS-CoV-2 RBD mutations that escape the monoclonal antibody LY-CoV555 and its cocktail with LY-CoV016. Cell Rep Med.

[17] Chen J, Wang R, Wang M, Wei GW (2020). Mutations Strengthened SARS-CoV-2 Infectivity. J Mol Biol.

[18] Cherian S, Potdar V, Jadhav S (2021). Convergent evolution of SARS-CoV-2 spike mutations, L452R, E484Q and P681R, in the second wave of COVID-19 in Maharashtra, India [PREPRINT]. Rapid Reviews COVID-19.

[19] (2021). WHO COVID-19 Weekly Epidemiological Update. https://apps.who.int/iris/bitstream/handle/10665/341525/CoV-weekly-sitrep25May21-eng.pdf?sequence=1.

[20] Campbell F, Archer B, Laurenson-Schafer H (2021). Increased transmissibility and global spread of SARS-CoV-2 variants of concern as at June 2021. Euro Surveill.

[21] Di Giacomo S, Mercatelli D, Rakhimov A, Giorgi FM (2021). Preliminary report on severe acute respiratory syndrome coronavirus 2 (SARS-CoV-2) Spike mutation T478K. J Med Virol.

[22] Xu C, Wang Y, Liu C (2021). Conformational dynamics of SARS-CoV-2 trimeric spike glycoprotein in complex with receptor ACE2 revealed by cryo-EM. Sci Adv.

[23] Shu Y, McCauley J (2017). GISAID: Global initiative on sharing all influenza data - from vision to reality. Euro Surveill.

[24] Guex N, Peitsch MC (1997). SWISS-MODEL and the Swiss-PdbViewer: an environment for comparative protein modeling. Electrophoresis.

[25] Krieger E, Vriend G (2014). YASARA View - molecular graphics for all devices - from smartphones to workstations. Bioinformatics.

[26] Yi C, Sun X, Ye J (2020). Key residues of the receptor binding motif in the spike protein of SARS-CoV-2 that interact with ACE2 and neutralizing antibodies. Cell Mol Immunol.

[27] Lee PI, Hu YL, Chen PY, Huang YC, Hsueh PR (2020). Are children less susceptible to COVID-19?. J Microbiol Immunol Infect.

[28] Zimmermann P, Curtis N (2021). Why is COVID-19 less severe in children? A review of the proposed mechanisms underlying the age-related difference in severity of SARS-CoV-2 infections. Arch Dis Child.

